# Models to Estimate Lactation Curves of Milk Yield and Somatic Cell Count in Dairy Cows at the Herd Level for the Use in Simulations and Predictive Models

**DOI:** 10.3389/fvets.2016.00115

**Published:** 2016-12-19

**Authors:** Kaare Græsbøll, Carsten Kirkeby, Søren Saxmose Nielsen, Tariq Halasa, Nils Toft, Lasse Engbo Christiansen

**Affiliations:** ^1^Dynamical Systems, Department of Applied Mathematics and Computer Science, Technical University of Denmark, Lyngby, Denmark; ^2^Section for Epidemiology, National Veterinary Institute, Technical University of Denmark, Frederiksberg, Denmark; ^3^Department of Large Animal Sciences, University of Copenhagen, Frederiksberg, Denmark

**Keywords:** milk yield, somatic cell count, dilution effect, production parameters, modeling, simulation, prediction, lactation curve

## Abstract

Typically, central milk recording data from dairy herds are recorded less than monthly. Over-fitting early in lactation periods is a challenge, which we explored in different ways by reducing the number of parameters needed to describe the milk yield and somatic cell count of individual cows. Furthermore, we investigated how the parameters of lactation models correlate between parities and from dam to offspring. The aim of the study was to provide simple and robust models for cow level milk yield and somatic cell count for fitting to sparse data to parameterize herd- and cow-specific simulation of dairy herds. Data from 610 Danish Holstein herds were used to determine parity traits in milk production regarding milk yield and somatic cell count of individual cows. Parity was stratified in first, second, and third and higher for milk, and first to sixth and higher for somatic cell count. Fitting of herd level parameters allowed for cow level lactation curves with three, two, or one parameters per lactation. Correlations of milk yield and somatic cell count were estimated between lactations and between dam and offspring. The shape of the lactation curves varied markedly between farms. The correlation between lactations for milk yield and somatic cell count was 0.2–0.6 and significant on more than 95% of farms. The variation in the daily milk yield was observed to be a source of variation to the somatic cell count, and the total somatic cell count was less correlated with the milk production than somatic cells per milliliter. A positive correlation was found between relative levels of the total somatic cell count and the milk yield. The variation of lactation and somatic cell count curves between farms highlights the importance of a herd level approach. The one-parameter per cow model using a herd level curve allows for estimating the cow production level from first the recording in the parity, while a two-parameter model requires more recordings for a credible estimate, but may more precisely predict persistence, and given the independence of parameters, these can be easily drawn for use in simulation models. We also conclude that using total somatic cell count may stabilize models, and therefore, the dilution factor is of importance in Danish Holstein.

## 1 Introduction

Productivity of the individual dairy cow is of central importance to dairy farmers: her milk production, reproductive performance, and somatic cell count (SCC). Whether we want to model a dairy farm or make accurate predictions of the future, an estimation of these traits is necessary. Predictions are important for making decisions on culling and replacements, and these have a substantial influence on the economy of the farm (1).

The daily variation in milk yield and SCC can be large even for healthy cows (2, 3). This large variation complicates certain future predictions which are of the utmost importance when making decisions on culling and replacement. It is, therefore, vital to create a robust estimate of milk yield and SCC to make accurate predictions. Longitudinal data with high variance have often been modeled using Bayesian methods such as the Kalman filter (4, 5). However, despite the modernization of the dairy industry by the use of, for example, automatic milking systems, data from the cow level in Denmark are most often recorded and stored on a monthly basis in the common milk recording systems. This sparse registration decreases the certainty of predictions, and it may, therefore, be important to decrease the number of parameters and the complexity of the methods describing the milk yield and SCC per cow, in order to increase the robustness of predictions.

Some of the most widely used lactation models to describe milk yield are by Wood (6) and Wilmink (7), and (in Denmark) Enevoldsen lactation curves (8–10). Most parametric mathematical lactation curves, including those mentioned, have parameters describing milk yield or energy corrected milk (ECM) as a function of days in milk (DIM), with an initial increase, a peak value or level, followed by a final decrease in milk yield. At least three parameters per lactation period are, therefore, required. Since the number of milk recordings per cow is low and the daily variation in yield is large (several kilos is not unusual), a robust fit of the lactation curve is rarely possible before the end of the lactation period. However, we hypothesize that the number of parameters needed to describe a lactation curve could be reduced by including information about all cows on the farm. When simulating a dairy herd in the most realistic way possible, an individual lactation curve must be assigned to every cow. Assigning an individual lactation curve means that the parameters describing such a lactation curve must be drawn from appropriate distributions (11). However, the parameters of most lactation curves are correlated (as we will demonstrate for the Wood curve). The solution can be to use a model where the parameters have no correlation. In this study, we derive two- and one-parameter models where the parameters have no correlation, and implement them on milk recordings.

The somatic cell count (SCC) is an indicator of mastitis (12–14) but is also influenced by other factors such as parity, breed, and DIM, which has been subjected to a large number of different parameterizations and statistical methods to parameterize (15–18). The SCC typically displays even larger variation than milk yield (change in order of magnitude). To handle the skewness of the variation and bring SCC to scale with milk yield, transformation of the measurement is required. Furthermore, the SCC is inversely proportional to the milk yield over time and in the single milk recording. This may be due to the dilution factor: the same amount of cells will give a lower cell count if the volume of milk is higher (19), and the fact that high yielding cows are more likely to have mastitis (20).

The objective of this paper was to determine the simplest robust models for cow level milk yield and SCC, so that fits can be made on sparse data. Furthermore, we investigate how the parameters typically change between parities, as well as the correlation between them. This information can be used to predict the most likely future value of the cow and to initialize the simulation of a dairy herd as realistically as possible given a typical dataset. To the best of our knowledge, this is the first study to show how robust predictions of milk production and SCC can be obtained using lactation curves with reduced number of parameters, or using un-correlated parameters for the lactation curves, which is useful in case of sparse data.

## 2 Materials and Methods

Data on milk production and SCC as well as demographic data were obtained from the Danish Cattle database (www.seges.dk), from which 610 herds with Holstein dairy cows were randomly selected among the approximately 3,000 Danish herds partaking in the regular milk recording. These herds were subjected to regular milk recording of all animals 6 or 11 times per year, including recording of milk yield, fat and protein content, and SCC. The most common was 11 recordings per year (in 91% of herds in the data set). All cows born before January 1, 2000 were excluded. The total data set included milk yield records from 293,929 individual cows. Data were further subset to include only the records collected between 6 and 305 (inclusive) days in milk (DIM). The latter limit was chosen to fit a standard 305-day lactation curve. The first 5 days were excluded due to a very large variation in the measurements observed from 0 to 5 DIM (data not shown). In total, 4,802,266 test day records with both milk yield and SCC count for the individual cows were present in the final data set. A minimum of 30 valid recordings per parity per farm were imposed to do the fitting. Here, valid means a positive number larger than 0, any recording not meeting this requirement on either milk or SCC was excluded. For milk, this reduced the number of farms included to 600, 594, and 587 when fitting first, second, and third and higher lactations. For the SCC, this reduced the number of farms included to 600, 594, 581, 568, 546, and 408, when fitting first to fifth and sixth and above lactations. The data contained no information regarding selectiveness of the testing, time of milking, production system, or number of milkings per day (all recordings are pooled daily). Further descriptive statistics can be found in Table 1.

**Table 1:** **Summary statistics of herds included in the study**.

	Median	Range (2.5–97.5%)	Unit
Years of data	11.6	3.4–11.9	Years
Lactating cows per recording date	80	1–268	No.
Lactating cows total	442	5–1,362	No.
Cows in lac. period 1	341	3–1,031	No.
Cows in lac. period 2	229	3–697	No.
Cows in lac. period ≥3	131	3–408	No.
Avr. age of lactating cows	3.9	3.1–5.5	Years
Total ECM lac. period 1	9,224	7,920–9,392	kg
Total ECM lac. period 2	10,750	9,094–10,934	kg
Total ECM lac. period ≥3	10,350	9,248–10,777	kg
Total SCC lac. period 1	551	355–882	mio.
Total SCC lac. period 2	979	519–1,627	mio.
Total SCC lac. period 3	1,322	692–2,124	mio.
Total SCC lac. period 4	1,566	845–2,866	mio.
Total SCC lac. period 5	1,716	865–3,706	mio.
Total SCC lac. period 6	1,899	794–24,127	mio.

*Total ECM (Energy Corrected Milk) and SCC (Somatic Cell Count) were found by summation of day to day estimates of ECM and tSCC (total Somatic Cell Count), which are calculated based on the one-parameter estimates, using a double log transformation for tSCC, over the 300-day period from days 6 to 305. The number of lactating animals is taken from the latest milking date*.

*mio., million; lac., lactation*.

In this paper, we speak generally of the SCC, but we use two distinct measures. The first is the usual SCC given by number per unit of milk per milliliter, which we will refer to as the unit somatic cell count (uSCC), and the second is the total number of somatic cells in the daily milk yield, which we refer to as total somatic cell count (tSCC). This measure is used to test whether the variation in milk yield accounts for some of the variation in the SCC. Furthermore, we introduce rECM and rtSCC where the “r” indicates that the measure is relative to a mean cow on a given farm. These measures express the relative value of milk or SCC compared to the average cow in a herd in the same stage of lactation and same parity. The relative measure is also equivalent to relative residuals of the fits to lactation and SCC curves.

### 2.1 Milk Yield

Milk yield was described as energy corrected milk (ECM) (21), which is defined as:

(1) ECM=milk(0.122 fat+0.077 protein+0.249)

where *milk* is milk in kilos, *protein* is protein in %, and *fat* is fat in %. A 305-day milk yield was fit to the standard three-parameter Wood’s curve for the first, second, and third lactation periods (6):

(2) $$f_{\mathrm{ijk}}^{\text{ ECM}}\left(\text{DIM}\right)=a_{\mathrm{ijk}}{\text{DIM}}^{b_{\mathrm{ijk}}}\text{ exp}\left(-\text{exp}\left(c_{\mathrm{ijk}}\right)\text{DIM}\right)$$

where parameter *a* is a scaling factor to represent yield at the beginning of lactation, and parameters *b* and *c* are factors associated with the inclining and declining slopes of the lactation curve, respectively, specific to lactation *j* of cow *i* on farm *k*. The *c* factor is exponentiated to achieve better scaling of the parameter when fitting and plotting. The Wood’s curve was selected because it displays consistently good performance with the fewest number of parameters (22), and it does not give negative values of milk yield for positive values of DIM. Parameters describing lactations were fitted for lactation periods 1, 2, and ≥3, as parameters typically do not change when fitting for higher lactations. For fitting individual Wood’s curves per cow, it was decided that at least six milk recordings were required per lactation per cow in order to initiate the fit. This requirement did not change the number of farms included per lactation, the number of individual cows included per farm can be found in Table 1.

**Figure 1:**
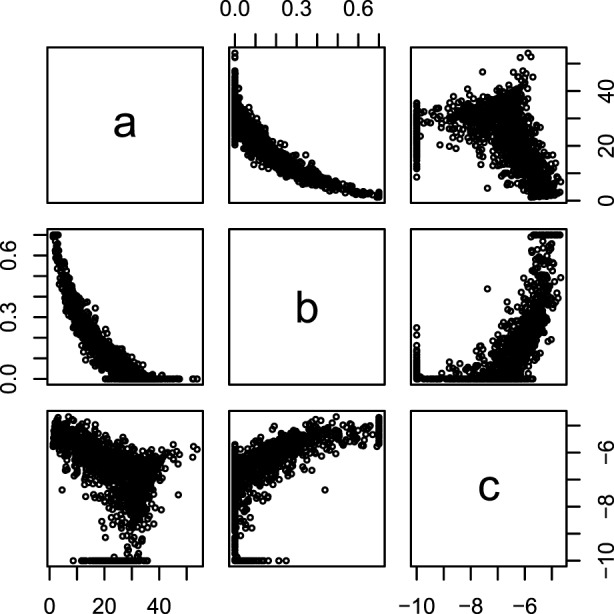
**Intercorrelation of Wood curve parameters**. The parameters of the Wood lactation curve [equation (2)] fitted to individual cows plotted against each other. Data from lactation one in one random farm.

The fitted parameters from equation (2) are plotted against each other for a single farm in Figure 1, which display correlation between the parameters of the lactation curves. From Figure 1, it was decided to use *b* as the predictor variable, because neither *a* ~ *c* nor *c* ~ *a* had a clear monotone correlation. These correlations can be parameterized as:

(3) $$f_{\mathrm{jk}}^{\;a}\left(b_{\mathrm{ij}}\right)=\theta_{1\mathrm{jk}}^{a}\text{ exp}\left(-\theta_{2\mathrm{jk}}^{a}b_{\mathrm{ij}}\right)$$

(4) $$f_{\mathrm{jk}}^{\;c}\left(b_{\mathrm{ij}}\right)=\theta_{1\mathrm{jk}}^{c}\text{ exp}\left(-\theta_{2\mathrm{jk}}^{c}b_{\mathrm{ij}}\right)+\theta_{3\mathrm{jk}}^{c}$$

where *f^ a^* and *f^ c^* are functions that describe the *a* and *c* parameters of the Wood curve as a function of *b* describing lactation *j* of cow *i* on farm *k*, given the five herd parameters $\theta_{1}^{a}$, $\theta_{2}^{a}$, $\theta_{1}^{c}$, $\theta_{2}^{c}$, and $\theta_{3}^{c}$. This allows us to describe each cow with two parameters instead of three:

(5) $$f_{\mathrm{ij}}^{\text{ ECM}}\left(\text{DIM},\alpha_{\mathrm{ij}},\beta_{\mathrm{ij}}\right)=\alpha_{\mathrm{ij}}N_{\mathrm{jk}}\left(\beta_{\mathrm{ij}}\right)f_{\mathrm{jk}}^{\;a}\left(\beta_{\mathrm{ij}}\right){\text{DIM}}^{\beta_{\mathrm{ij}}}\text{ exp}\left(-\text{exp}\left(f_{\mathrm{jk}}^{\;c}\left(\beta_{\mathrm{ij}}\right)\right)\text{DIM}\right)$$

(6) $$N_{\mathrm{jk}}\left(\beta \right)=\frac{\sum_{i}^{i\text{ in }\mathrm{jk}}\sum_{\hat{\text{DIM}}=6}^{305}\;a_{\mathrm{ijk}}{\hat{\text{DIM}}}^{b_{\mathrm{ijk}}}\text{ exp}\left(-\text{exp}\left(c_{\mathrm{ijk}}\right)\hat{\text{DIM}}\right)}{\sum_{\hat{\text{DIM}}=6}^{305}\;f_{\mathrm{jk}}^{\;a}\left(\beta \right){\hat{\text{DIM}}}^{\beta }\text{ exp}\left(-\text{exp}\left(f_{\mathrm{jk}}^{\;c}\left(\beta \right)\right)\hat{\text{DIM}}\right)}$$

where *β*_*ij*_ is the shape parameter of lactation *j* of cow *i*, given information about all cows at farm *k*, *N_jk_*(*β*_*ij*_) is a normalizing function where the double sum in the numerator is equivalent to the farm average milk production in the given lactation, so that when fitting *α*_*ij*_ and *β*_*ij*_ in equation (5), the *α*_*ij*_ becomes the milk level of the individual cow *i* compared to the average 305-day lactation yield on farm *k* in lactation *j*.

Fitting the *θ* parameters in functions *f^ a^*(*b*) and *f^ c^*(*b*) included comparing the area under curve (AUC) of Wood’s curve using the parameters *b*, *f^ a^*(*b*), and *f^ c^*(*b*) to the AUC calculated from the originally fitted *a*, *b*, and *c* parameters per cow. Implemented by writing a custom made function that calculates the sum of squared relative residuals of equations (3) and (4) and the AUC. This function was minimized over the *θ*s using nlminb in the statistical open source program R version 3.1.1 (23). The fitting of the *α* and *β* was done thereafter using the nls function also in R.

A one-parameter model per cow can be described as:

(7) $${\hat{f}}_{\mathrm{ij}}^{\text{ECM}}\left(\text{DIM},\alpha_{\mathrm{ij}}^{M}\right)=\alpha_{\mathrm{ij}}^{M}{\hat{a}}_{\mathrm{jk}}{\text{DIM}}^{{\hat{b}}_{\mathrm{jk}}}\text{ exp}\left(-\text{exp}\left({\hat{c}}_{\mathrm{jk}}\right)\text{DIM}\right)$$

where each cow, *i*, is defined by a milk yield level $\alpha_{\mathrm{ij}}^{M}$ that is proportional to the average cow in lactation *j* on farm *k*, given the parameters ${\hat{a}}_{\mathrm{jk}}$, ${\hat{b}}_{\mathrm{jk}}$, and ${\hat{c}}_{\mathrm{jk}}$. The superscript *M* refers to the milk yield, later we shall introduce the superscript *C* which refers to the SCC.

A relative lifetime yield for each cow relative to the farm average cow was calculated by taking all milk recordings per cow and takes the mean of the values of these relative to the average cow [equation (7) with *α* = 1]. The relative lifetime yield was then compared between generations (dams and offspring) by correlation analysis.

### 2.2 Somatic Cell Count

We tested two different transformations on both uSCC and tSCC, to best model the SCC. This consisted of two count transformations: a log and double log transformation of the count data; each observing either the SCC per ml (uSCC) or the total amount of cells delivered by the cow during milking (tSCC), which is given as: tSCC = uSCC · *kg*.*milk*.

After transformations, the data were fitted using a Wilmink style curve (7). SCC was chosen to be parameterized as a Wilmink style curve, because SCC typically starts high and quickly goes toward lower values, after which it becomes constant or slowly rising. These properties are inherent to the Wilmink curve, but for lactation curves the parameters have the opposite sign:

(8) $$T\left(\text{uSCC}\cdot t+\Delta_{T}\right)\left(\text{DIM}\right)={\tilde{a}}_{\mathrm{jk}}+{\tilde{b}}_{\mathrm{jk}}\text{ DIM}+\text{exp}\left(-\text{exp}\left({\tilde{c}}_{\mathrm{jk}}\right)\text{DIM}\right){\tilde{d}}_{\mathrm{jk}}$$

where $\tilde{a}$, $\tilde{b}$, $\tilde{c}$, and $\;\tilde{d}$ are the parameters describing the SCC of lactation *j* on farm *k*, and *T* represents either a log or a log–log transformation, with the corresponding offset Δ*_T_* being 1 for the log transform or exp(1) for the double log transform, and *t* is either 1 for the unit uSCC or *kg.milk* for the tSCC.

When fitting a one-parameter model of the SCC we used:

(9) $$\text{log}\left(\text{log}\left(\text{tSCC}+\text{exp}\left(1\right)\right)\right)\left(\text{DIM},\alpha_{\mathrm{ij}}^{C}\right)=\alpha_{\mathrm{ij}}^{C}\left({\tilde{a}}_{\mathrm{jk}}+{\tilde{b}}_{\mathrm{jk}}\text{ DIM}\right.\left.+\text{ exp}\left(-\text{exp}\left({\tilde{c}}_{\mathrm{jk}}\right)\text{DIM}\right){\tilde{d}}_{\mathrm{jk}}\right)$$

where $\alpha_{\mathrm{ij}}^{C}$ is the level of somatic cells produced for each cow *i* relative to the log–log transformed average in lactation *j* on farm *k*.

A relative lifetime SCC for each cow relative to the farm average cow was calculated by taking all SCC recordings per cow and takes the mean of the values of these relative to the average cow [equation (9) with *α*^*C*^ = 1]. The relative lifetime SCC was then compared between generations (dams and offspring) by correlation analysis.

The normality of residuals for the SCC was assessed visually by Quantile–Quantile (Q–Q) plots. The variance was found according to the statistical definition, it is also approximately equal to the slope of the lines plotted in the Q–Q plot.

### 2.3 Correlations

Correlation and linear dependency between fitted parameters were tested using the cor.test() and lm() functions in the statistical open source program R version 3.1.1 “Sock It to Me” (23). Local Polynomial Regression Fitting was done using loess() also in R.

## 3 Results

**Figure 2:**
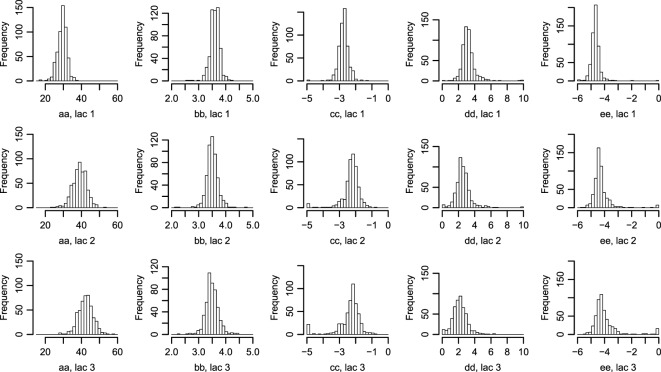
**Histograms of parameters describing the correlation between Wood curve parameters**. Using equations (3) and (4), the *θ* parameters describing the correlation seen in Figure 1 are fitted for the farms. (The labels in the figure correspond to the parameters in the equations as such: $\text{aa}=\theta_{\text{1}}^{\text{a}}$, $\text{bb}=\theta_{\text{2}}^{\text{a}}$, $\text{cc}=\theta_{\text{1}}^{\text{c}}$, $\text{dd}=\theta_{\text{2}}^{\text{c}}$, $\text{ee}=\theta_{\text{3}}^{\text{c}}$.) Abbreviation: lac., lactation.

**Figure 3:**
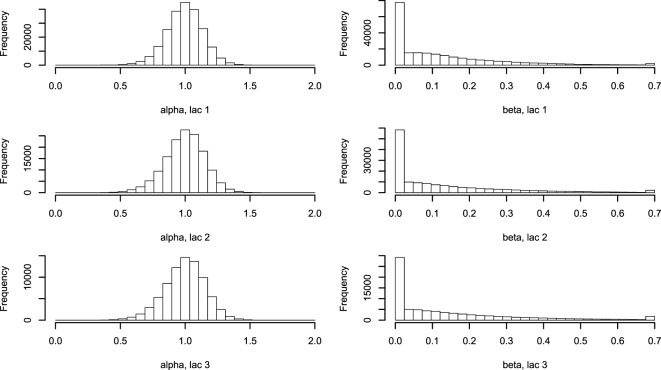
**Histogram of *α* and *β* describing the two-parameter lactation curve**. Using equations (3) and (5), lactation curves with only two parameters per cow were fitted for all cows for lactations 1, 2, and ≥3. Abbreviation: lac., lactation.

**Figure 4:**
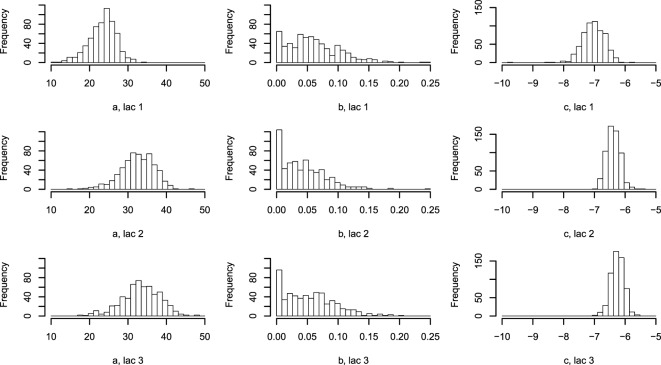
**Histogram of Wood curve parameters fitted per lactation per farm**. Parameters describing the average lactation curve per lactation per farm [equation (7)]. Abbreviation: lac., lactation.

**Figure 5:**
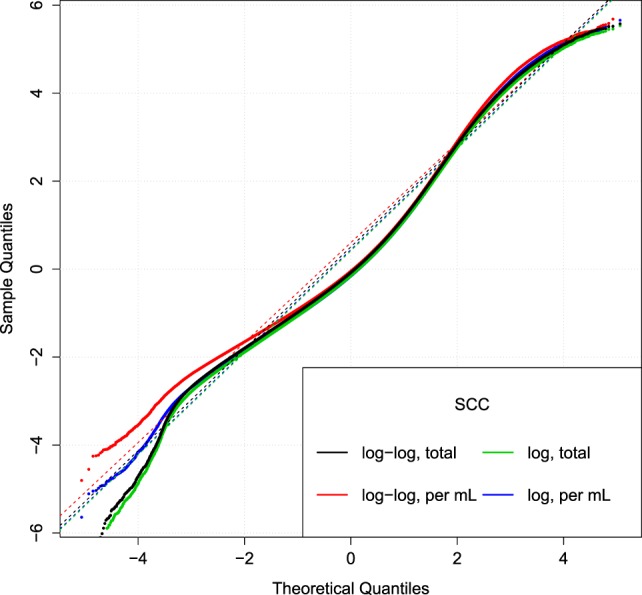
**Comparison of SCC models**. Q–Q plot of the relative residuals after using two different transformations (log or log–log) combined with the total (tSCC) or per ml SCC (uSCC) [equation (9)]. Broken lines are Q–Q lines using the 95% range.

The data generally showed large variances in milk yield and SCC across animals and farms (see Figures 1–4; Tables 1–3), and a large variation around the milk or SCC predicted levels (i.e., Figure 5).

**Table 2:** **Median herd parameters of lactation and tSCC (total Somatic Cell Count)**.

tSCC	$\tilde{\text{a}}$	$\tilde{\text{b}}$	$\tilde{\text{c}}$	$\tilde{\text{d}}$	–
Lac. 1	1.99	1.13e − 04	−2.18	0.211	
Lac. 2	2.08	6.65e − 05	−1.87	0.171	
Lac. 3	2.12	4.58e − 05	−1.55	0.233	
Lac. 4	2.14	2.49e − 05	−1.35	0.305	
Lac. 5	2.15	1.32e − 05	−1.27	0.333	
Lac. 6	2.16	7.11e − 06	−1.35	0.290	

**Lactation curves**	$\theta_{1}^{a}$	$\theta_{2}^{a}$	$\theta_{1}^{c}$	$\theta_{2}^{c}$	$\theta_{3}^{c}$

Lac. 1	30.3	3.66	−2.43	3.24	−4.94
Lac. 2	39.8	3.44	−1.79	3.69	−4.87
Lac. ≥3	42.5	3.38	−1.68	3.58	−4.84

**Figure 6:**
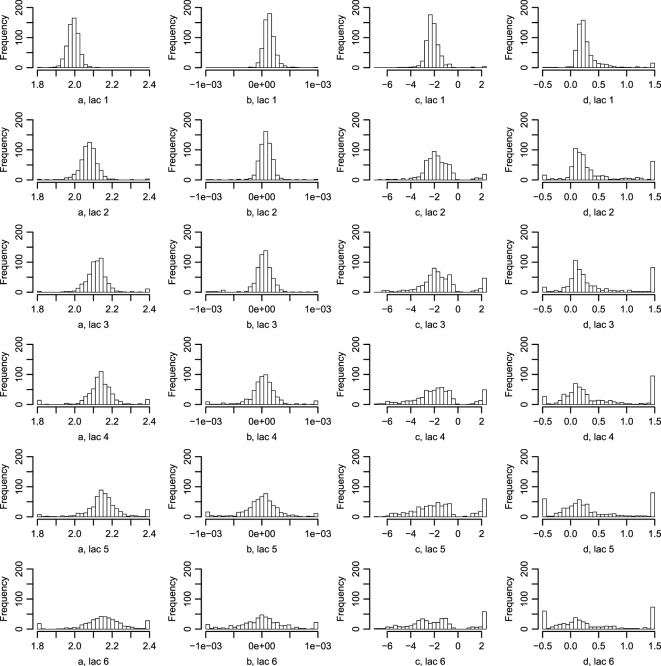
**Histograms of the parameters describing the tSCC**. Using equation (9), the tSCC was fitted independently for each farm for lactation 1–6. Abbreviation: lac., lactation.

*From equations (3), (4), and (9) depicted in Figures 2 and 6*.

*lac., lactation*.

**Table 3:** **Correlation coefficients of ECM (Energy Corrected Milk) and tSCC (total Somatic Cell Count)**.

	Direction (*i–j*)^a^	Correlation	P value	Range (2.5–97.5%)
ECM	1–2	0.44	<0.001	0.23–0.64
ECM	2–3	0.42	<0.001	0.12–0.62
ECM	1–3	0.31	<0.001	0.09–0.54
ECM dam–offspring	*L–L*	0.11	<0.001	−0.06 to 0.30
tSCC	1–2	0.39	<0.001	0.22–0.57
tSCC	2–3	0.40	<0.001	0.21–0.62
tSCC	1–3	0.27	<0.001	0.10–0.47
tSCC dam–offspring	*L–L*	0.14	<0.001	−0.03 to 0.31

*^a^Direction (*i*–*j*) refers to the correlation between lactation number *i* and *j* (i.e., 1−2 refers to the correlation of the levels between the first and the second lactation), subscript *L* refers to relative lifetime value. Correlation values are global values, while ranges are for the individual farms*.

### 3.1 Milk Yield

Fitting standard Wood lactation curves to all lactation periods of all cows resulted in correlation structures which are exemplified in Figure 1. From this, we observed that parameter *b* had the most consistent correlation structure with *a* and *c*, and this was then parameterized using equations (3) and (4).

The fitted correlation structure for all farms in the data set gave the derived parameters *θ* (Figure 2), which are normally distributed within farms and stable over lactation periods, except for $\theta_{1}^{a}$ which seemingly accounts for most of the difference in milk yield across lactations.

The five farm parameters, *θ*, can then be used to inform a two-parameter model for each cow [equation (5)], the results of which can be seen in Figure 3. Milk yield levels, *α*, were normally distributed. The *β* estimates have a large proportion of zeros, which correspond to a straight-line lactation curve with no initial increase. The *β* estimates were fitted to an exponential distribution giving a (mean) rate λ of 6.74. The median parameters of *θ* are listed in Table 2.

The one-parameter model, with one lactation curve per lactation per farm described by three parameters each and one scale parameter per cow, shows large differences in the parametrization between farms (Figure 4), while the scale parameters for each cow around the average per farm are normally distributed (not shown).

### 3.2 Somatic Cell Count

Figure 5 displays the Q–Q plot of relative residuals when fitting the SCC using four combinations of transformations. The residuals are close to a straight line indicating a good fit. The similar curvature of the Q–Q plots indicates that the transformations are of similar goodness.

**Figure 7:**
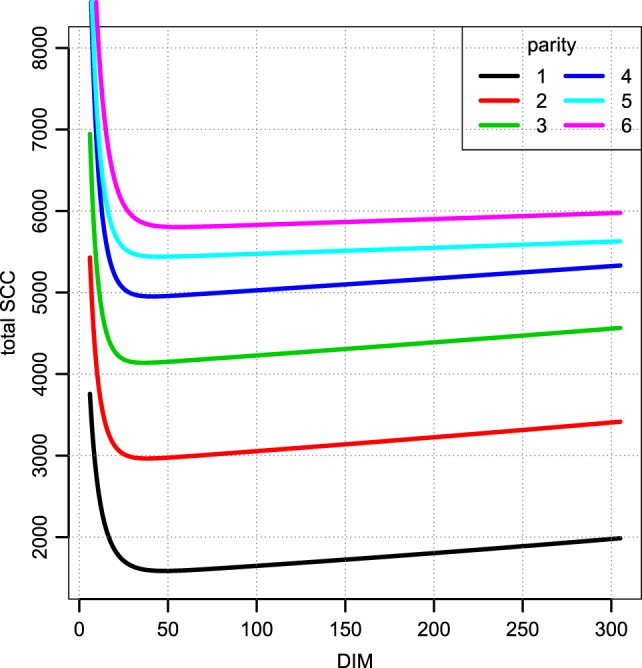
**Total SCC as per the median parameters fitted per farm**. Median of parameters from Figure 6. Notice that this value must be divided by the daily milk yield if it is to be compared to a SCC measured in counts per ml. The unit is million counts.

Figure 6 displays the fitted values to equation (9) for each of the farms that met the inclusion criteria (30 recordings per parity). The parameters of the tSCC do not intercorrelate (not shown). The median values were used to make Figure 7, which show how the predicted tSCC increased with parity. The SD of *α*^*C*^, which describes cows’ tSCC level compared to the average cow on a farm was 0.051.

**Figure 8:**
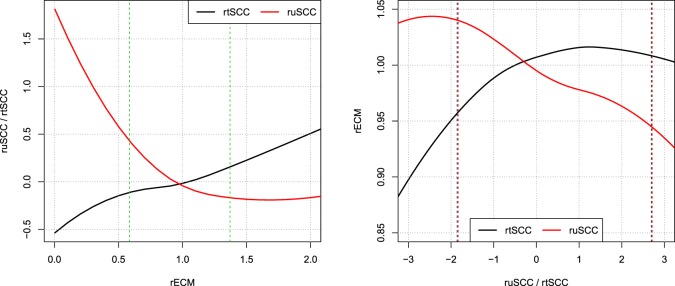
**Correlations of relative residuals rECM and ruSCC/rtSCC**. Loess smoothing of the relative residuals [i.e., observed ECM/predicted ECM and log(observed SCC/predicted SCC)]. Showing the correlations of ECM and SCC. Vertical dashed lines represent the interval wherein 95% of data resides.

Figure 8 shows local polynomial regression fitting of relative residuals when predicting ECM and SCC using both the tSCC and uSCC. The figure shows that there is stronger correlation between the relative residuals of uSCC and ECM, than between tSCC and ECM. This leads to two different interpretations: the uSCC curve indicates that higher SCC reduces the milk yield, and the rtSCC curve indicates no correlation when the increase happens above the average. Figure 8 displays the log transform of tSCC, but the log–log transformation gives identical results within the 95% data interval (not shown).

### 3.3 Correlations

The correlation of relative milk yield levels, *α*^*M*^, between lactations was above 0.3 when using the one-parameter model on all cows (see Table 3). The variation in correlations on farm level was generally low.

The overall correlation in lifetime production of ECM between dams and their offspring compared to the farm average was 0.11 (P < 0.001). This hereditary trait was, however, not found to be significant on all farms (Table 3).

The correlations of the tSCC showed almost identical numbers to the correlations of the ECM (Table 3).

The level of the *α*^*C*^ for tSCC as function of *α*^*M*^ for ECM for first lactation animals has an overall correlation of 0.18 when considering all values, but only 0.02 when considering the 50% of cows producing milk around the average (Table 3).

## 4 Discussion

The parameters provided in this paper describe milk yield and SCC-specific traits of Danish Holstein cows required for modeling a functional dairy farm. We have specifically opted to fit our functions, so that we can include the variation in a simulation model. Variance is an important factor given that we wish to make decisions based on the differences between individual cows.

Results of simulation models are often prone to considerable variation due to the use of a large number of parameters depicted from stochastic distributions (24). This large variation may affect how willing farmers, veterinarians, and/or decision makers are in accepting these results, and hence limit their practical application in the field. It is, therefore, important to seek ways of reducing this variation by, for instance, increasing the precision of the parameters. This will hopefully increase the confidence in model results and motivate farmers to apply recommendations based on these results.

### 4.1 Milk Yield

We have here tested two new ways of parameterizing the lactation curve of dairy cows with a reduced number of parameters. Both methods are farm specific, and the results clearly show large variation in the yield and the shape of lactation curves between farms. It is, therefore, always prudent to adjust for the effect of each farm when modeling across a population. Our two parameterizations include three or five parameters per farm per lactation period describing the herd, which enables the number of parameters describing the milk yield level of the individual cow to be reduced to one or two values, respectively. The fewer the cow level parameters the earlier in a lactation a fit of lactation curve can be performed. However, if the shape is of importance (i.e., to make inference about the persistence) two parameters per cow are a minimum.

Reducing the number of lactation curve parameters allows for fitting of parameters in early lactation for individual cows, when few measurements have been made. Furthermore, we can introduce parameters that are directly relevant to culling and replacement decisions, such as the milk yield level relative to the farm average (*α* and *α*^*M*^) or the shape factor (*β*) describing the shape of the curve. The type of models presented in this manuscript further has the advantage of a unique form of the lactation curve for each parity on each farm compared to, i.e., fixed and random effects models where the effect is only on the total yield. The differences in the shape of the curves are likely due to both genetic differences and management practices, but as we do not have access to these data, using unique lactation curves per farm is a possible way of capturing the effects of these unknown parameters.

Several different lactation curves have previously been described and used [for a recent comparison see, e.g., Ref. (22)]. However, to the best of our knowledge, this is the first time that lactation curves with a reduced number of parameters have been used to eliminate correlations between parameters of the lactation curves. Reducing the number of parameters allows for prediction with fewer milk recordings per cow (e.g., early in lactation), as some information is already obtained by fitting herd level parameters. Eliminating correlation of parameters allows for sampling from independent distributions when simulating cows.

Negative values of *β* would result in high milk yields at low and high DIM (i.e., an inverse lactation curve). When fitting, *β* can then be pushed toward a negative value for certain combinations of milk recordings in the early lactation. To avoid this, we restricted *β* to be positive or zero. This restriction is likely not necessary when working with daily milk yield recordings (e.g., milking robot data). Preliminary results from a small sample of robot data indicate that the *β* parameter may be more normally distributed around a positive non-zero value when sufficient data are present (data not shown).

We also performed a parameterization similar to equation (7) using a Wilmink lactation curve (7) (results not shown), but the Wilmink curve does not perform well when the lactation curve is close to a straight line. In this case, two of the four parameters will become completely indeterminable, with only their sum being fixed. For this reason, an average or median parameter cannot be established, as the value of these parameters depends on the specific fitting algorithm and starting values used.

### 4.2 Somatic Cell Count

Parameters describing SCC were fitted per farm and for parity 1, 2, …, ≥6, as parameters typically do not change when fitting for higher parities (data not shown).

We have shown that using the total count of somatic cells in the milk is less correlated with the milk yield compared to the measure of SCC per milliliter. This indicates that for healthy milking cows, the production of somatic cells in the udder may be detached of the daily milk production. The observed drop in uSCC may be attributed to the dilution effect also reported by Green et al. (19). The dilution effect causes high-yielding cows to have lower SCC per ml than low-yielding animals in the same herd and with the same infection status. The use of total SCC would be most robust as it may prevent premature culling of lower-yielding cows due to their presumed high SCC. However, other studies have found no effect of dilution (25). Indeed, various results are reported in literature; most report some form of negative correlation between milk yield and SCC, but many only above a certain cut-off (10, 13–17, 26–28). The association between milk yield and SCC in this manuscript concerns mainly healthy cows with normal SCC levels. We speculate that the dilution factor is of importance to cows with SCC below a cutoff, which might indicate some form of disease, likely mastitis.

A few observations call for a note. We have not excluded cows based on possible (subclinical) mastitis status, so an increase in the prevalence of mastitis in later parities may be the reason for the increase seen in Figure 7. However, there is no discernable separation of the *α*^*C*^ levels into two groups (results not shown). Extreme values in Figures 5 and 8 represent very low SCC values (approximately <5,000 cells/ml). These values account for around 0.1% of the data. It is not common to have cows with such low SCC and most importantly, SCC will not be the reason for culling such cows.

### 4.3 Correlations

Correlation structures of milk yield and SCC are important in order to build realistic simulation models of dairy herds. If all cows were the same, any small reduction of production parameters in a cow resulting from, e.g., disease may mark this cow for culling. Including individual cow parameters makes it possible to assess the value at which a cow becomes less worth than their herd mates and should hence be replaced. Such a model will not only be cow specific but also herd specific, and decisions can be made on individual cows in specific herds.

The correlations that we observe are consistent regardless of the transformation chosen, meaning that we are free to choose whichever transformation of the tested that best fulfills our assumptions of a good model.

The correlation structures that we observe in Figure 8 reveal that a higher milk yield compared to the average cow on a farm leads to a decrease in uSCC production, which is a clear indication of a dilution effect. In comparison, the tSCC is less correlated. When performing the local polynomial smoothing (loess) on the ECM as a function of SCC, the effect of increased tSCC is negligible for residual values above the predicted value. For values below the mean, the linear correlation comes from number of cells being low, and the tSCC is then driven by milk production, which gives the linear correlation between rECM and rtSCC.

We have not investigated the effect of sire on the milk yield, which is beyond the scope of the project at this stage. We have previously investigated the effect of sex of the offspring, which was found to be small compared to between farm effects (29). There exists a large body of literature on hereditary effects in dairy cows, in which correlation structures between cow-specific traits are studied [e.g., Ref. (30–33)]. However, we have mainly concerned this paper with the correlations between parities of the same cow, so that we in simulations and predictive models have an estimate for how current knowledge may translate to future production. Health related parameters are scarcely registered in the national registry and require proper evaluation before inclusion in predictive models.

## 5 Conclusion

We have presented two new ways of reducing the number of parameters to describe the lactation curves for the individual cow. These methods were developed with a robust prediction tool in mind, so that estimations can be carried out with as few data points as possible, and therefore as early as possible in the lactation period. We observed that in addition to the amount of ECM produced per year, the shape of the lactation curves also varied significantly between herds, which may be of great importance when predicting future yields.

Furthermore, we demonstrated that the SCC was less correlated with milk production when using the total production of cells per day. This emphasizes that the dilution factor is of importance, and we recommend that future predictions be based on the tSCC.

Finally, we presented correlation structures for ECM and tSCC between lactations. Overall milk and tSCC were highly correlated between lactations of the individual cow, both on the individual farm and on average over all farms, which makes predictions of future values possible.

## Author Contributions

Developed models, performed data analysis, and wrote the manuscript: KG. Assisted in developing of models, analysis of data, and drafting of the manuscript: LC. Assisted in analysis of data and drafting of the manuscript: CK, SN, TH, and NT.

## Conflict of Interest Statement

The authors declare that the research was conducted in the absence of any commercial or financial relationships that could be construed as a potential conflict of interest. The reviewer NS-d-R and handling Editor declared their shared affiliation, and the handling Editor states that the process nevertheless met the standards of a fair and objective review.

## References

[1] Kristensen E Østergaard S Krogh MA Enevoldsen C. Technical indicators of financial performance in the dairy herd. J Dairy Sci (2008) 91:620–31. 10.3168/jds.2007-0201 18218749

[2] Schutz M Hansen L Steuernagel G Kuck A Variation of milk, fat, protein, and somatic cells for dairy cattle. J Dairy Sci (1990) 73:484–93. 10.3168/jds.S0022-0302(90)78697-3

[3] Schepers A Lam T Schukken Y Wilmink J Hanekamp W. Estimation of variance components for somatic cell counts to determine thresholds for uninfected quarters. J Dairy Sci (1997) 80:1833–40. 10.3168/jds.S0022-0302(97)76118-6 9276824

[4] Goodall E Sprevak D A bayesian estimation of the lactation curve of a dairy cow. Anim Prod (1985) 40:189–93. 10.1017/S0003356100025290

[5] Swalve H. Theoretical basis and computational methods for different test-day genetic evaluation methods. J Dairy Sci (2000) 83:1115–24. 10.3168/jds.S0022-0302(00)74977-0 10821588

[6] Wood P Algebraic model of the lactation curve in cattle. Nature (1967) 216:164–5. 10.1038/216164a0

[7] Wilmink J Comparison of different methods of predicting 305-day milk yield using means calculated from within-herd lactation curves. Livest Prod Sci (1987) 17:1–17. 10.1016/0301-6226(87)90049-2

[8] Enevoldsen C Elgaard K Jensen N Nielsen K Nørregaard H Philipsen J Identification of interactions and non-linear effects, assessment of herd effects, and model validation in a dairy herd health management context. Proceedings of the Ninth ISVEE Breckenridge, CO, USA: The International Society for Veterinary Epidemiology and Economics (2000). p. 1133–5.

[9] Nielsen SS Enevoldsen C Gröhn YT. The *Mycobacterium avium* subsp. *paratuberculosis* elisa response by parity and stage of lactation. Prev Vet Med (2002) 54:1–10. 10.1016/S0167-5877(02)00008-9 12062515

[10] Bennedsgaard TW Enevoldsen C Thamsborg S Vaarst M. Effect of mastitis treatment and somatic cell counts on milk yield in Danish organic dairy cows. J Dairy Sci (2003) 86:3174–83. 10.3168/jds.S0022-0302(03)73920-4 14594237

[11] Kirkeby C Graesbøll K Nielsen S Christiansen L Toft N Rattenborg E Simulating the epidemiological and economic impact of paratuberculosis control actions in dairy cattle. Front Vet Sci (2016) 3:90. 10.3389/fvets.2016.00090 27777933 PMC5056316

[12] Hortet P Seegers H. Calculated milk production losses associated with elevated somatic cell counts in dairy cows: review and critical discussion. Vet Res (1998) 29:497–510. 9851006

[13] de Haas Y Barkema HW Veerkamp RF. The effect of pathogen-specific clinical mastitis on the lactation curve for somatic cell count. J Dairy Sci (2002) 85:1314–23. 10.3168/jds.S0022-0302(02)74196-9 12086069

[14] Halasa T Nielen M De Roos A Van Hoorne R de Jong G Lam T Production loss due to new subclinical mastitis in dutch dairy cows estimated with a test-day model. J Dairy Sci (2009) 92:599–606. 10.3168/jds.2008-1564 19164670

[15] Harris B Winkelman A Test-day model for national genetic evaluation of somatic cell count in New Zealand. Proceedings of the 2004 Interbull Meeting, Vol. 101; Sousse, Tunisia (2004).

[16] Robert-Granié C Foulley J-L Maza E Rupp R Statistical analysis of somatic cell scores via mixed model methodology for longitudinal data. Anim Res (2004) 53:259–73. 10.1051/animres:2004016

[17] Piwczynski D Sitkowska B Statistical modelling of somatic cell counts using the classification tree technique. Arch Tierz (2012) 55:332–45.

[18] Archer SC Mc Coy F Wapenaar W Green MJ. Association of season and herd size with somatic cell count for cows in Irish, English, and Welsh dairy herds. Vet J (2013) 196:515–21. 10.1016/j.tvjl.2012.12.004 23321453

[19] Green LE Schukken Y Green M. On distinguishing cause and consequence: do high somatic cell counts lead to lower milk yield or does high milk yield lead to lower somatic cell count? Prev Vet Med (2006) 76:74–89. 10.1016/j.prevetmed.2006.04.012 16780974

[20] Piepers S Schukken YH Passchyn P De Vliegher S. The effect of intramammary infection with coagulase-negative staphylococci in early lactating heifers on milk yield throughout first lactation revisited. J Dairy Sci (2013) 96:5095–105. 10.3168/jds.2013-6644 23769365

[21] Sjaunja L Baevre L Junkkarinen L Pedersen J Setälä J A nordic proposal for an energy corrected milk (ECM) formula. Performance recording of animals: state of the art. Proceedings of the 27th Biennial Session of the International Committee for Animal Recording (ICAR) (Vol. 50). Paris: The International Committee for Animal Recording (1990). p. 156–7.

[22] Adediran S Ratkowsky D Donaghy D Malau-Aduli A. Comparative evaluation of a new lactation curve model for pasture-based Holstein-Friesian dairy cows. J Dairy Sci (2012) 95:5344–56. 10.3168/jds.2011-4663 22916941

[23] R Development Core Team. R: A Language and Environment for Statistical Computing. Vienna, Austria: R Foundation for Statistical Computing (2014).

[24] Robinson S Simulation: The Practice of Model Development and Use. Houndmills: Palgrave Macmillan (2014).

[25] Boland F OGrady L More S. Investigating a dilution effect between somatic cell count and milk yield and estimating milk production losses in Irish dairy cattle. J Dairy Sci (2013) 96:1477–84. 10.3168/jds.2012-6025 23295120

[26] Tyler JW Thurmond MC Lasslo L. Relationship between test-day measures of somatic cell count and milk production in California dairy cows. Can J Vet Res (1989) 53:182. 2713782 PMC1255545

[27] Bartlett PC Miller GY Anderson CR Kirk JH. Milk production and somatic cell count in michigan dairy herds. J Dairy Sci (1990) 73:2794–800. 10.3168/jds.S0022-0302(90)78966-7 2283411

[28] Hand K Godkin A Kelton D. Milk production and somatic cell counts: a cow-level analysis. J Dairy Sci (2012) 95:1358–62. 10.3168/jds.2011-4927 22365217

[29] Græsbøll K Kirkeby C Nielsen SS Christiansen LE. Danish Holsteins favor bull offspring: biased milk production as a function of fetal sex, and calving difficulty. PLoS One (2015) 10:e0124051. 10.1371/journal.pone.0124051 25874441 PMC4395430

[30] Berry D Buckley F Dillon P Evans R Rath M Veerkamp R. Genetic relationships among body condition score, body weight, milk yield, and fertility in dairy cows. J Dairy Sci (2003) 86:2193–204. 10.3168/jds.S0022-0302(03)73809-0 12836956

[31] Berry DP Buckley F Dillon P Evans RD Veerkamp RF Genetic relationships among linear type traits, milk yield, body weight, fertility and somatic cell count in primiparous dairy cows. Ir J Agric Food Res (2004) 43:161–76.

[32] Carlén E Strandberg E Roth A. Genetic parameters for clinical mastitis, somatic cell score, and production in the first three lactations of Swedish Holstein cows. J Dairy Sci (2004) 87:3062–70. 10.3168/jds.S0022-0302(04)73439-6 15375069

[33] Pritchard T Coffey M Mrode R Wall E. Genetic parameters for production, health, fertility and longevity traits in dairy cows. Animal (2013) 7:34–46. 10.1017/S1751731112001401 23031504

